# Histamine H4 Receptor Antagonist Ameliorates the Progression of Experimental Autoimmune Encephalomyelitis via Regulation of T-Cell Imbalance

**DOI:** 10.3390/ijms242015273

**Published:** 2023-10-17

**Authors:** Abdullah A. Aldossari, Mohammed A. Assiri, Mushtaq A. Ansari, Ahmed Nadeem, Sabry M. Attia, Saleh A. Bakheet, Thamer H. Albekairi, Hatun A. Alomar, Haneen A. Al-Mazroua, Taghreed N. Almanaa, Mohammed A. Al-Hamamah, Mohammad Y. Alwetaid, Sheikh F. Ahmad

**Affiliations:** 1 Department of Pharmacology and Toxicology, College of Pharmacy, King Saud University, Riyadh 11451, Saudi Arabia; 2 Department of Botany and Microbiology, College of Science, King Saud University, Riyadh 11451, Saudi Arabia

**Keywords:** H4R antagonist, JNJ 10191584, EAE, multiple sclerosis, inflammatory mediators transcription factors

## Abstract

Multiple sclerosis (MS) is a degenerative condition characterized by immune-mediated attacks on the central nervous system (CNS), resulting in demyelination and recurring T-cell responses. The histamine H4 receptor (H4R) is mainly expressed in cellular populations and plays a vital role in inflammation and immunological responses. The role of H4R in neurons of the CNS has recently been revealed. However, the precise role of H4R in neuronal function remains inadequately understood. The objective of this work was to investigate the impact of JNJ 10191584 (JNJ), a highly effective and specific H4R antagonist, on the development of experimental autoimmune encephalomyelitis (EAE) and to gain insight into the underlying mechanism involved. In this study, we examined the potential impact of JNJ therapy on the course of EAE in SJL/J mice. EAE mice were administered an oral dose of JNJ at a concentration of 6 mg/kg once a day, starting from day 10 and continuing until day 42. Afterward, the mice’s clinical scores were assessed. In this study, we conducted additional research to examine the impact of JNJ on several types of immune cells, specifically Th1 (IFN-γ and T-bet), Th9 (IL-9 and IRF4), Th17 (IL-17A and RORγt), and regulatory T (Tregs; Foxp3 and TGF-β1) cells in the spleen. In this study, we further investigated the impact of JNJ on the mRNA expression levels of *IFN-γ*, *T-bet*, *IL-9*, *IRF4*, *IL-17A*, *RORγt, Foxp3*, and *TGF-β1* in the brain. Daily treatment of JNJ effectively reduced the development of EAE in mice. The percentages of CD4^+^IFN-γ^+^, CD4^+^T-bet^+^, CD4^+^IL-9^+^, CD4^+^IRF4^+^, CD4^+^IL-17A^+^, and CD4^+^RORγt^+^ cells were shown to decrease, whereas the percentages of CD4^+^TGF-β1^+^ and CD4^+^Foxp3^+^ cells were observed to increase in EAE mice treated with JNJ. Therefore, the HR4 antagonist positively affected the course of EAE by modulating the signaling of transcription factors. The identified results include possible ramifications in the context of MS treatment.

## 1. Introduction

Multiple sclerosis (MS) is a neuroinflammatory disease of the central nervous system (CNS) characterized by demyelinating plaques that arise from axonal and myelin sheath damage [1,2]. It affects the brain, spinal cord, and optic nerves. The clinical manifestations of MS are characterized by inflammatory demyelination, infiltration of immune cells, and the release of inflammatory mediators [3,4]. A growing body of research indicates the compromised functionality of many resistant system components in MS patients. Experimental autoimmune encephalomyelitis (EAE) serves as a model for MS in humans. This model involves the induction of autoreactive T helper cells responsible for causing inflammation and demyelination in the CNS. EAE is frequently used in research to investigate the immunopathological mechanisms underlying MS and to explore potential therapeutic interventions. Prior research has elucidated the involvement of T cells in the pathogenesis of MS and EAE [5,6]. The role of T lymphocytes in beginning and driving disease pathogenesis is of utmost importance [7]. It is hypothesized that a subset of T lymphocytes undergo activation in the peripheral regions, traverse the blood-brain barrier, induces inflammation in the CNS, and interferes with neurotransmission [8,9]. The pathogenesis of MS is additionally influenced by T cells, which release proinflammatory cytokines. This process results in inflammatory infiltrates, demyelination, and axonal damage inside the EAE [10]. Furthermore, it has been observed that the functionality of regulatory T cells, which play a crucial role in regulating inflammation and the immune system, is diminished in individuals with MS and EAE [11,12].

While other immune cell types are involved in the development of EAE [13], the study’s emphasis on MS primarily revolves around CD4^+^ T cells due to their significant involvement in neuroinflammation in both MS and EAE. MS, a condition characterized by inflammation, is influenced by Th1 cells that express IFN-γ [14]. These Th1 cells, a specific subset of CD4^+^ T cells, play a crucial role in initiating the development of EAE [15,16]. The secretion of cytokines and specific cell surface markers are distinguishing features of these Th1 cells [17]. Mice lacking T-bet transcription factors demonstrated resistance to the induction of EAE [18]. Similarly, Gocke et al. [19] found that inhibiting T-bet signaling in a mouse model of EAE inhibited the development of the disease. The study demonstrated that Th1 cells have a role in promoting the infiltration of Th17 cells into the central CNS and triggering EAE [20]. Furthermore, Th9 cells and their secreted cytokine IL-9 play a significant role in activating T-cells during autoimmune inflammation of the central CNS [21]. These cells have been implicated in MS, collaborating with other CD4^+^ T cells and CNS-resident cells in MS and EAE [22,23]. This study demonstrates that interleukin-9 (IL-9) plays a crucial role in facilitating the migration of Th17 cells into the CNS. This migration is mediated by the production of CC chemokine ligand-20 (CCL20) by astrocytes [24]. The study showed that inhibiting interferon regulatory factor 4 (IRF4), a protein expressed in lymphocytes, macrophages, and dendritic cells, decreased EAE scores [25].

The involvement of Th17 cells in the pathogenesis of MS has been well documented in scientific literature [26]. Furthermore, the transplantation of Th17 cells has been found to elicit atypical EAE, as evidenced by the study conducted by Stromnes et al. [27]. Furthermore, the relative abundance of Th17 cells within the population of CD4^+^ T cells is regarded as a reliable measure of the extent of CNS inflammation in EAE mice [28]. The regulation of Th17 cells is mediated by the expression of the RORγt transcription factor [29]. Th17 cells expressing RORγt and T-bet simultaneously and producing IL-17A and IFN-γ exhibit a heightened level of pathogenicity and are selectively attracted to the CNS. This observation suggests that the T-bet plays a role in augmenting the pathogenic properties of Th17 cells [16,19]. Elevated levels of IL-17 have been observed in individuals with MS during periods of active disease [30]. Furthermore, the expression of IL-17 is purportedly controlled by Th9 cells generated by plasmacytoid dendritic cells, as reported [31]. On the other hand, regulatory T cells (Tregs), the primary immunosuppressive T cells, positively impact EAE by inhibiting the immune response to myelin-specific Th1 cells and shifting the Th1/Th2 balance towards Th2 cells. Consequently, treating Tregs has been shown to confer protection against EAE in mice [32,33]. It has been established that TGF-β-induced Tregs can suppress autoimmune disorders [34]. Furthermore, previous studies have emphasized the correlation between Tregs and the improvement of EAE [35]. Additionally, it has been observed that Tregs originating from the CNS substantially diminish the manifestations of EAE [36].

The histamine H4 receptor (H4R) is a recently identified G protein-coupled receptor subfamily member [37,38]. Its role in inflammatory illnesses and immunomodulatory pathways has been investigated [39,40]. The expression of H4R can be observed in several immune cells, such as mast cells, eosinophils, T cells, dendritic cells, and basophils. H4R modulates various functions in these cells, including activation, migration, and the generation of cytokines and chemokines [41,42,43]. There has been growing interest in pharmacological research about the potential use of H4R in treating immune-related illnesses [44]. The study demonstrated the significant involvement of H4R in neuroinflammation and immunological diseases [45]. The functional expression of H4R has been observed in neurons inside the CNS [46]. Furthermore, Strakhova et al. [47] identified the presence of H4R in the CNS of both humans and rodents. The expression of this gene is mainly observed in the cerebellum, with significantly lower levels detected in the hippocampus [48]. The expression of this gene has been verified in monocytes and neurons inside brain slices [49]. Dunford and colleagues provided further insights into the established impacts of histamine on the immune system and emphasized the potential therapeutic applications of H4R antagonists [50]. According to Coruzzi et al. [51], utilizing H4R antagonists has proven effective in treating inflammation. H4 receptor antagonists exert anti-inflammatory effects by inhibiting cytokine and chemokine production and modulating transcription factor signaling pathways [52,53]. The H4R antagonist JNJ 10191584 demonstrates high affinity and selectivity for both human and rodent H4R [54]. The anti-inflammatory properties of JNJ 10191584 were investigated in a study that included an experimental colitis model [55].

EAE is widely recognized as the prevailing preclinical model employed for investigating MS and has played a pivotal role in advancing the discovery of disease-modifying medicines in clinical contexts [56]. Inflammatory demyelinating lesions distinguish EAE localized explicitly in the CNS [57]. The most prevalent presentations of early MS in humans are relapse and remission [58]. This pattern is also observed in SJL/J mice through the induction of the proteolipid protein (PLP_139–151_)-induced relapsing-remitting (RR) form of MS (RR-MS) [59,60,61]. Hence, the EAE model generated by PLP is well-suited for evaluating prospective therapeutic interventions for RR-MS [5,62,63]. There are multiple pharmaceutical options accessible for the treatment of patients diagnosed with MS, yet the effectiveness of these therapeutic interventions is still considered inadequate. The present study aimed to evaluate the effect of the H4R antagonist, JNJ 10191584, in an EAE mouse model. We hypothesized that JNJ could alleviate EAE by regulating Th1/Th9/Th17/Tregs transcription factor signaling.

## 2. Results

### 2.1. The Administration of JNJ Demonstrates an Improvement in the Clinical Score of EAE

**Figure 1:**
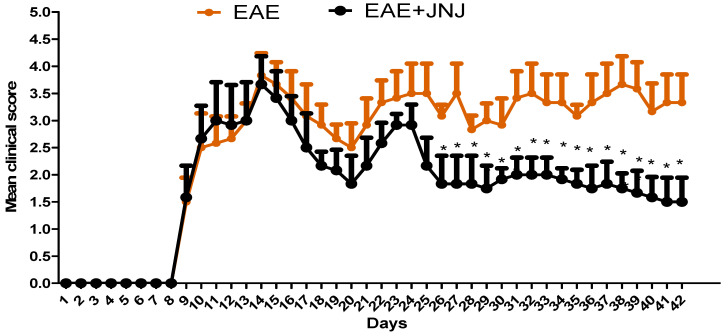
Illustrates the impact of JNJ on the clinical score of SJL/J mice inoculated with PLP_139–151_ to produce EAE. The graph depicts the illness scores observed in mice with EAE treated with either saline or JNJ. Naive SJL/J mice received saline treatment, while EAE mice were also treated with saline. Additionally, EAE mice were subjected to a treatment of 6 mg/kg, p.o. JNJ, once daily from day 10 to day 42, resulting in the EAE+JNJ group. All data are presented as mean ± SD (n = 6). Statistical significance was set at * *p* < 0.05.

To evaluate the hypothesis that JNJ has a protective impact on EAE, we administered JNJ treatment to mice with EAE. The findings of our study indicate that the administration of JNJ treatment resulted in a significant improvement in the severity of the disease in mice with EAE compared to mice who received a vehicle treatment (Figure 1). The findings of our study indicate that the administration of JNJ has a therapeutic impact in mitigating the severity of RR-EAE. These results also imply that H4R antagonists have promise as prospective therapeutic agents for treating EAE.

### 2.2. The Administration of JNJ Suppresses the Signaling Pathway of IFN-γ/T-Bet in Mice with EAE

**Figure 2:**
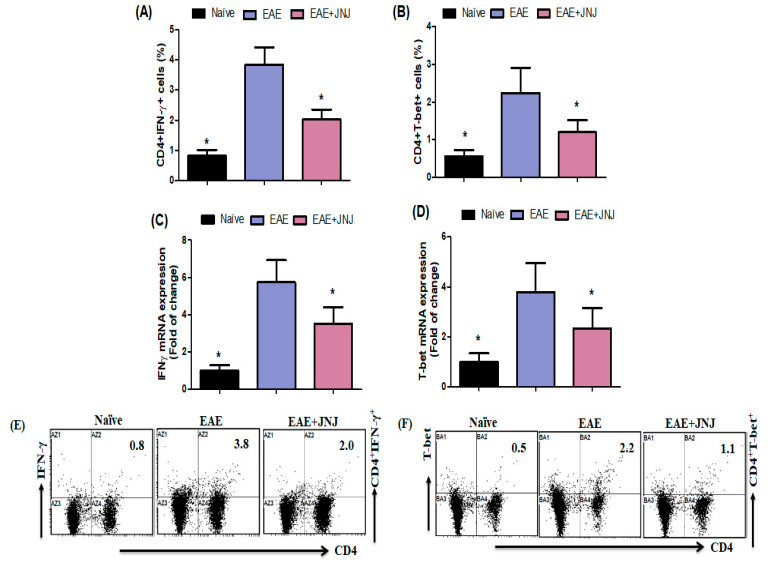
(**A**,**B**). The impact of JNJ on CD4^+^ T cells expressing IFN-γ and T-bet in the spleen was assessed by flow cytometry. (**C**,**D**) The effect of JNJ on the levels of *IFN-γ* and *T-bet* mRNA expression in the brain was evaluated using RT-PCR. The analysis focused on determining the proportion of Th1 cells within the CD4^+^ subset. This was achieved using intracellular labeling of IFN-γ and T-bet. (**E**,**F**) The resulting flow cytometry dot plots visually represented the alterations in expression levels of CD4^+^IFN-γ^+^ and CD4^+^T-bet^+^ in spleen cells. Naive SJL/J mice received saline treatment, while EAE mice were also treated with saline. Additionally, EAE mice were subjected to a treatment of 6 mg/kg, p.o. JNJ, once daily from day 10 to day 42, resulting in the EAE+JNJ group. All data are presented as mean ± SD (n = 6). Statistical significance was set at * *p* < 0.05.

To examine the potential anti-inflammatory properties of JNJ in EAE, we evaluated the levels of IFN-γ and T-bet expression in the spleen cells and brains of EAE animals. Flow cytometry analysis revealed a significant reduction in the proportion of CD4^+^ T cells expressing IFN-γ and T-bet in the spleen of mice with EAE following treatment with JNJ, as compared to the group treated with the vehicle control (Figure 2A,B). As determined by RT-PCR, the mRNA expression levels for *IFN-γ* and *T-bet* decreased in the brains of EAE mice treated with JNJ compared to EAE animals treated with the vehicle (Figure 2C,D). The administration of JNJ suppressed the upregulation of IFN-γ and T-bet expression, indicating its potential as an anti-inflammatory agent. Figure 2E,F display dot plots illustrating CD4^+^IFN-γ^+^ and CD4^+^T-bet^+^ cells, respectively, as observed using flow cytometry analysis. Our research outcomes suggest that JNJ exerted a notable inhibitory effect on Th1 cells in mice with EAE.

### 2.3. JNJ Exhibits Inhibitory Effects on the Signaling Pathway of Th9-Related Transcription Factors in EAE

**Figure 3:**
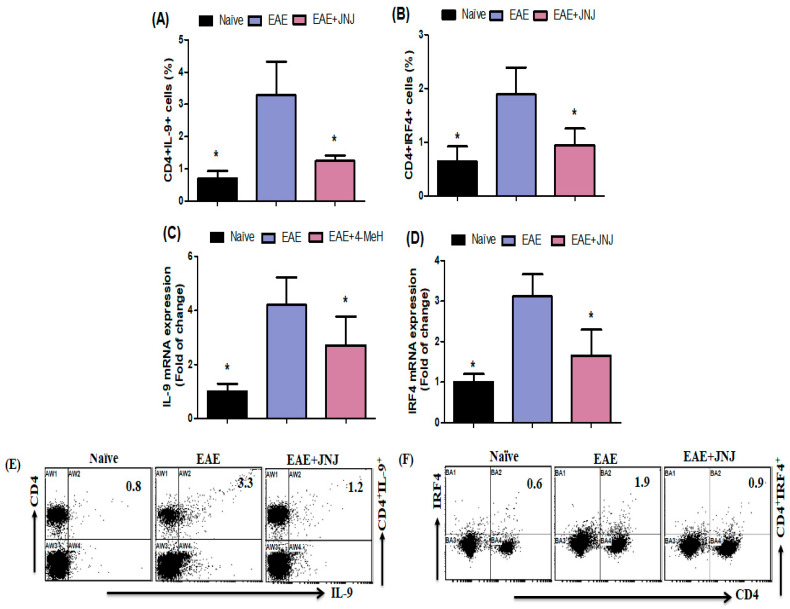
(**A**,**B**) The impact of JNJ on CD4^+^ T cells expressing IL-9 and IRF4 in the spleen was assessed by flow cytometry. (**C**,**D**) The effect of JNJ on *IL-9* and *IRF4* mRNA expression levels in the brain was evaluated using RT-PCR. Quantifying Th9 cells within the CD4^+^ subset by utilizing intracellular labeling techniques for IL-9 and IRF4. (**E**,**F**) The alterations in expression levels of CD4^+^IL-9^+^ and CD4^+^IRF4^+^ in spleen cells were visually represented using typical flow cytometry dot plots. Naive SJL/J mice received saline treatment, while EAE mice were also treated with saline. Additionally, EAE mice were subjected to a treatment of 6 mg/kg, p.o. JNJ, once daily from day 10 to day 42, resulting in the EAE+JNJ group. All data are presented as mean ± SD (n = 6). Statistical significance was set at * *p* < 0.05.

To explore the underlying processes responsible for the alleviation induced by JNJ in mice with EAE, we investigated the impact of JNJ on Th9 cells. It was observed that the population of CD4^+^ T cells expressing IL-9 and IRF4 was elevated in the spleens of EAE mice treated with a vehicle. Conversely, the EAE animals treated with JNJ exhibited a notable reduction in CD4^+^ T cells expressing IL-9 and IRF4 (Figure 3A,B). To gain a deeper understanding of the molecular mechanism of JNJ, we investigated alterations in mRNA expression levels of IL-9 and IRF4 in the brain through the utilization of RT-PCR. It was observed that the expression of IL-9 and IRF4 was markedly elevated in mice with EAE compared to EAE mice treated with a control vehicle. In contrast, the mice with EAE treated with JNJ exhibited a notable reduction in *IL-9* and *IRF4* mRNA levels in the brain, as depicted in Figure 3C,D. Figure 3E,F display representative dot plots illustrating the presence of CD4^+^IL-9^+^ and CD4^+^IRF4^+^ cells, respectively. The data presented in this study suggest that Th9 cells are a specific target of JNJ and play a crucial role in mediating the therapeutic effects of JNJ in the EAE model.

### 2.4. JNJ Inhibits Th17 Signaling in EAE

**Figure 4:**
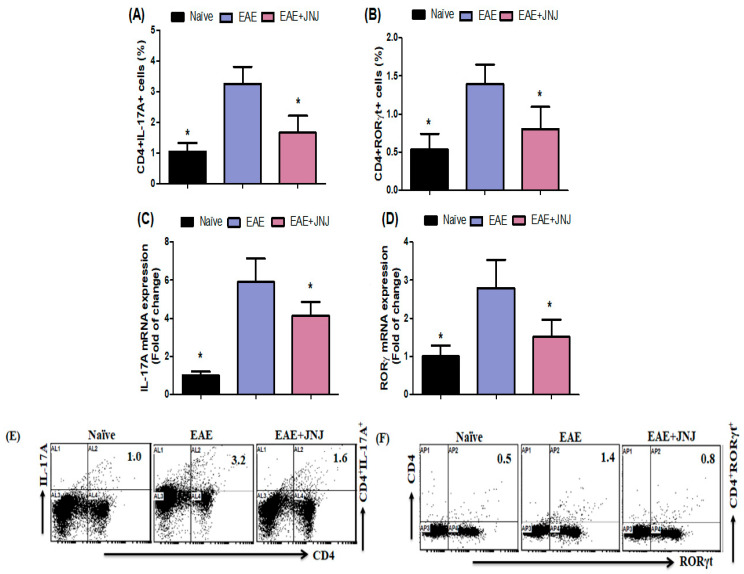
(**A**,**B**) The impact of JNJ on CD4^+^ T cells expressing IL-17A and RORγt in the spleen was assessed by flow cytometry. (**C**,**D**) The analysis of the effect of JNJ on *IL-17A* and *RORγ* mRNA expression levels in the brain was conducted using the RT-PCR method. The quantification of Th17 cells within the CD4^+^ subset was accomplished by utilizing intracellular labeling techniques targeting IL-17A and RORγt. (**E**,**F**) The alterations in expression levels of CD4^+^IL-17A^+^ and CD4^+^RORγt^+^ in spleen cells were visually represented using typical flow cytometry dot plots. Naive SJL/J mice received saline treatment, while EAE mice were also treated with saline. Additionally, EAE mice were subjected to a treatment of 6 mg/kg, p.o. JNJ, once daily from day 10 to day 42, resulting in the EAE+JNJ group. All data are presented as mean ± SD (n = 6). Statistical significance was set at * *p* < 0.05.

To examine the anti-inflammatory properties of JNJ in EAE, we assessed the presence of CD4^+^ T cells expressing IL-17A and RORγt in the spleens of EAE mice. According to the data presented in Figure 4A,B, there was a notable increase in the CD4^+^ T cells expressing IL-17A and RORγt in mice with EAE treated with a vehicle compared to the control group (NC group). Conversely, the number of Th17 cells was dramatically reduced in EAE animals treated with JNJ (Figure 4A,B). This study assessed IL-17A and RORγ mRNA expression in the brain. The mRNA expression levels of *IL-17A* and *RORγ* were consistently shown to be significantly reduced in JNJ-treated animals with EAE compared to mice treated with the vehicle (Figure 4C,D). Figure 4E,F display dot plots illustrating CD4^+^IL-17A^+^ and CD4^+^RORγt^+^ cells, respectively, as observed using flow cytometry analysis. Therefore, the findings of our study indicate that the administration of JNJ effectively suppressed Th17 signaling in mice with EAE, thereby demonstrating its potential as a possible therapeutic intervention for MS.

### 2.5. The Administration of JNJ Enhances the Activation of Regulatory T Cell (Treg) Signaling in Mice with EAE

**Figure 5:**
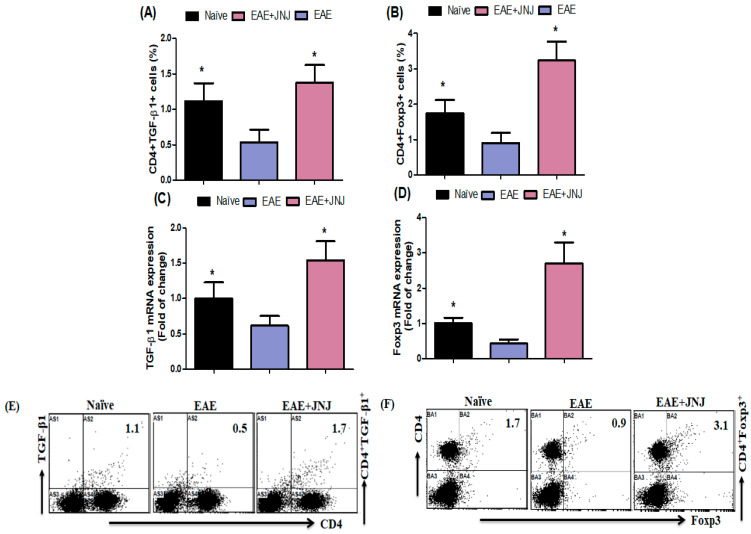
(**A**,**B**) The impact of JNJ on CD4^+^ T cells expressing TGF-β1- and Foxp3 in the spleen was assessed by flow cytometry. (**C**,**D**) The effect of JNJ on the expression levels of *TGF-β1* and *Foxp3* mRNA in the brain was evaluated using RT-PCR. The quantification of Tregs within the CD4^+^ subset was accomplished by utilizing intracellular labeling techniques targeting TGF-β1 and Foxp3. (**E**,**F**) The alterations in the expression levels of CD4^+^TGF-β1^+^ and CD4^+^Foxp3^+^ in spleen cells were visually represented using typical flow cytometry dot plots. Naive SJL/J mice received saline treatment, while EAE mice were also treated with saline. Additionally, EAE mice were subjected to a treatment of 6 mg/kg, p.o. JNJ, once daily from day 10 to day 42, resulting in the EAE+JNJ group. All data are presented as mean ± SD (n = 6). Statistical significance was set at * *p* < 0.05.

We conducted additional investigations to determine whether JNJ regulates the production of TGF-β1 and Foxp3, a crucial transcription factor associated with Tregs. The mice with EAE treated with JNJ had a notably elevated proportion of CD4^+^ T cells expressing TGF-β1 and Foxp3 in the spleen, as depicted in Figure 5A and Figure 4B. To gain a deeper understanding of the mechanism of JNJ, RT-PCR was used to quantify the mRNA expression levels of *TGF-β1* and *Foxp3* in the brains of the experimental mice (See Supplementary Materials). The mRNA levels of TGF-β1 and Foxp3 were considerably elevated in mice with EAE treated with JNJ compared to mice treated with a vehicle (Figure 5C and Figure 4D). Figure 5E,F depicts example dot plots obtained using flow cytometry, illustrating the presence of CD4^+^TGF-β1^+^ and CD4^+^Foxp3^+^ cells. The findings of this study suggest that the treatment of JNJ resulted in an increase in the population of Tregs in mice with EAE.

## 3. Discussion

MS is a chronic neurological condition characterized by potentially severe long-term consequences. EAE is a CNS demyelinating illness caused by CD4^+^ T-cells. It is commonly employed as an animal model for the investigation of MS. The involvement of autoreactive CD4^+^ T cells in the onset and coordination of EAE has been well-documented in previous studies [64,65]. Activated CD4^+^ T lymphocytes undergo migration from peripheral tissues to the CNS, where they trigger an inflammatory cascade through the production of cytokines and chemokines [66]. T cells are believed to significantly impact the progression of EAE [67]. Prajeeth et al. [68] also observed that T cells release inflammatory mediators that activate microglia. The study conducted by Yang et al. [16] demonstrated that T lymphocytes play a role in disease progression in the CNS. The EAE model in mice involves the administration of myelin autoimmune antigens, which lead to axonal injury. This injury is a consequence of an immune response that is not regulated correctly, ultimately resulting in clinical symptoms that resemble those observed in individuals with MS, including paralysis and problems in gait [5,69].

A prior investigation documented the functional manifestation of H4R in both human and rodent neurons, emphasizing its significance in neuronal functioning. The expression of H4R has been reported in the peripheral nerves and neurons of the submucosal plexus [70,71]. The presence of H4R has been documented in several regions of the CNS, including the hippocampus, thalamus, amygdala, cortex, striatum, and spinal cord [46,47]. An electrophysiological method study demonstrated that H4R directly hyperpolarizes cortical neurons [47]. A subsequent investigation into the putative functions of H4R in the human brain, utilizing its anticipated functional associations within the human proteome, proposed the involvement of H4R in regulating circadian rhythms and suppressing neuronal activity [72]. The primary objective of this investigation was to investigate the therapeutic potential of an H4R antagonist in the context of EAE, a well-established murine model of MS characterized by persistent inflammation and demyelination. Our findings indicate that the administration of JNJ therapy significantly reduced the clinical symptoms observed in mice with EAE. Hence, JNJ exhibits a therapeutic impact on RR-EAE, resulting in a decrease in both the severity and progression of the disease. The findings of this study suggest that the use of H4R antagonists has the potential to mitigate the progression of symptoms in EAE. Consequently, the results of our study indicate that H4R antagonists represent innovative therapeutic targets for the treatment of MS.

The function of Th1 cells in the pathogenesis of EAE has been identified as crucial [13]. Furthermore, these cells are linked to the formation of inflammatory EAE lesions in the CNS [73]. The potential of IFN-γ as a biomarker for the progression of MS has been highlighted [74]. It was discovered that individuals with MS had a higher concentration of CD4^+^ T cells expressing IFN-γ and T-bet in both the brain and CSF fluid [75]. It also implicated IFN-γ in developing MS and the EAE mice model [76]. Moreover, it has been observed that animals lacking T-bet exhibit resistance to the induction of EAE, providing further evidence for a correlation between Th1 cells and CSN autoimmunity [18]. The current investigation showed that the quantity of CD4^+^ T cells expressing IFN-γ and T-bet in mice with EAE treated with JNJ was comparatively reduced compared to mice with EAE treated with the vehicle. Additionally, the treatment with JNJ reduced IFN-γ and T-bet mRNA levels in the brains of mice with EAE. Therefore, the results of our study indicate that the administration of JNJ therapy effectively hinders the course of EAE by suppressing the Th1 immune response. The findings of this study provide additional evidence supporting the therapeutic efficacy of JNJ in EAE mice. Moreover, the findings elucidate the correlation between JNJ and the Th1 pathway in EAE mice, establishing a foundation for the preclinical assessment of H4R antagonist therapy for MS. These results suggest that H4R antagonists could be an innovative therapeutic focus for MS.

Multiple recent reports have indicated the potential contribution of Th9 cells to the development and progression of MS. There is a growing body of evidence suggesting that Th9 cells play a role in the induction of EAE and contribute to the promotion of inflammation in the CNS [13]. A further investigation substantiated that mice with a deficiency in IL-9 exhibited a safeguard against the onset of EAE [21]. The literature has documented the impact of IL-9 on individuals diagnosed with MS [31]. The study conducted by Ouyang et al. [77] demonstrated a significant association between levels of IL-9 and the severity of systemic lupus erythematosus, indicating a potential pivotal function of IL-9 in the development and progression of this disease. The effective suppression of IL-17 production by the anti-IL-9 monoclonal antibody (mAb) indicates that IL-9 may serve as a viable target for inhibiting Th17 cells, which have been implicated in the pathogenesis of EAE [24]. Previous studies have reported the significant involvement of IRF4 in the developmental processes of T and B cells [78]. The suppression of IRF4 hinders the development of Th1/Th17 cells, improving EAE [79]. The current study observed more CD4^+^ T cells expressing IL-9 and IRF4 in mice with EAE than in those treated with JNJ. Furthermore, the mRNA levels of IL-9 and IRF4 were shown to be elevated in mice with EAE. However, mice with EAE treated with JNJ had considerably reduced expression levels of these genes. Therefore, the results obtained from our study indicate a potential association between the amelioration of EAE in mice treated with JNJ. Our investigation’s findings support the potential therapeutic efficacy of the H4R antagonist in promoting EAE in the context of MS.

The expression of IL-17A, a molecular marker associated with Th17 cells, is observed in a wide range of CNS lesions, CSF, and blood samples obtained from individuals diagnosed with MS [80]. The migration of Th17 cells specific to myelin occurs within the CNS. These cells release IL-17A, which induces the production of chemokines. These chemokines recruit a range of immune cells, emphasizing myeloid cells, to the CNS. This process initiates and sustains an inflammatory cascade [69]. The study conducted by Arellano et al. [81] established a positive correlation between demyelinating lesions in the CNS in MS and EAE and elevated levels of IFN-γ and IL-17. This finding suggests that these cytokines may play a significant role in the development and progression of MS. Relapse, akin to the onset of disease, is distinguished by the accumulation of Th17 immune responses [25]. According to Balasa et al. [82], the incidence of recurrence and relapse rates can be decreased by targeting Th17 cells. The increased expression of IL-17 in T cells and glial cells that infiltrate the CNS is linked to the development of more severe MS [83]. In the current investigation, the administration of JNJ decreased the quantity of CD4^+^ cells expressing IL-17A and RORγt in the spleens of mice with EAE. Notably, the administration of JNJ considerably suppressed the production of proinflammatory factors IL-17A and RORγt in mice with EAE. The findings of this study provide evidence that JNJ displays anti-inflammatory properties in an EAE mice model. Our study’s findings indicate that JNJ can decrease the activity of Th17 cells in mice with RR-EAE. This observation may explain the positive outcomes observed in RR-EAE using H4R antagonists.

There is a reported correlation between an elevated occurrence of Treg cells and a reduction in the quantity of inflammatory cells, namely inflammatory myeloid cells. These inflammatory myeloid cells are known to be the predominant cell types responsible for inflicting damage to the CNS in EAE and MS. According to Giles et al. [84], Machado-Santos et al. [85], and Ramaglia et al. [86], the findings of the study demonstrated that TGF-β exhibited a reduction in demyelination, viral antigen expression, and macrophage recruitment in a murine model of MS. TGF-β, a very influential modulator of the immune system, exerts a neuroprotective function in the context of MS [87,88]. This study demonstrates that the induction of Foxp3 by TGF-β hinders the development of Th17 cells by counteracting the action of RORγt [89,90]. The conclusive demonstration of Tregs participation in the active regulation of neuroinflammatory processes in EAE [91,92]. Prior research has demonstrated the role of TGF-β in regulating autoimmune reactions and its possible therapeutic advantages in mitigating inflammatory harm in individuals with MS. Furthermore, TGF-β in mice exhibited enhanced EAE outcomes by influencing the function of natural killer cells [93,94]. Dysregulation of Foxp3 expression gives rise to various autoimmune disorders, such as MS [95]. A correlation exists between the decrease in Foxp3 expression and the advancement of disease in MS [96]. The present investigation showed that mice with EAE treated with JNJ exhibited notable augmentation in the spleen of the CD4^+^ T cells expressing TGF-β1 and Foxp3. Additionally, there was a substantial elevation in the brain’s amounts of TGF-β1 and Foxp3 mRNA. Hence, the observed decrease in the severity of EAE following JNJ treatment in these mice can be ascribed to the upregulation of the anti-inflammatory signaling pathways. The findings of our study suggest that H4R antagonists have a previously unidentified function in modulating the imbalance of T-cells in the EAE mice model.

In conclusion, the administration of JNJ demonstrated improvement in the clinical symptoms of EAE in mice. Furthermore, it was observed that JNJ showed inhibitory effects on the Th1, Th9, and Th17 inflammatory signaling pathways, while concurrently inducing the upregulation of Tregs in the EAE animal model. The results of our study emphasize the therapeutic efficacy of JNJ in addressing autoimmune disorders, specifically MS. The findings present novel preclinical evidence supporting the potential use of H4R antagonists as a therapeutic approach for MS.

## 4. Materials and Methods

### 4.1. Reagents

The potent and selective H4R antagonist JNJ 10191584 (JNJ), phorbol myristate acetate, ionomycin, and RPMI medium were purchased from Sigma-Aldrich (St. Louis, MO, USA). Golgi-plug, anti-CD4 FITC (#100406), anti-CD4 PE (#100408), anti-CD4 APC (#100412), anti-CD4 PE/Dazzle (#100566), anti-IFN-γ APC/Cyanine7 (#505850), anti-T-bet PE (#644810), anti-IL-9 PE (#514104), anti-IRF4 Alexa Fluor^®^ 488 (#646406), anti-IL-17A PE/Dazzle (#506938), anti-RORγt PE (#562607), anti-TGF-β1 PE (#141404), and anti-Foxp3 PE (#320008) mouse monoclonal antibodies, as well as RBC lysing solution, permeabilization solution, and fixation buffers, were purchased from BioLegend and BD Biosciences (San Diego, CA, USA). The primers were purchased from GenScript (Piscataway, NJ, USA). TRIzol was purchased from Invitrogen (Thermo-Fisher Scientific, Carlsbad, CA, USA). High-capacity cDNA and SYBR Green kits were purchased from Applied Biosystems (Foster City, CA, USA).

### 4.2. Animals

Female SJL/J mice, aged 8–10 weeks and weighing 25–30 g, were purchased from Jackson Laboratories (Bar Harbor, ME, USA). Mice were housed per cage in an appropriate environment, maintained in a 12 h light/dark cycle, and given ad libitum access to food and water. The mice were allowed to acclimatize for 2–3 weeks before initiation of the experiment. All experimental animal protocols were approved by the King Saud University Animal Scientific Research Ethical Committee (Ethical Approval No: KSU-SE-21-50).

### 4.3. EAE Induction and JNJ Administration

To establish the RR-EAE model, SJL/J mice were subcutaneously immunized with 200 µg of PLP_139–151_ peptide emulsified with complete Freund’s adjuvant (Hooke Laboratories, Lawrence, MA, USA) at four sites (50 μL/site). Each mouse received 200 ng pertussis toxin (Hooke Laboratories) intraperitoneally (i.p.) on the day of immunization [61,97,98]. To evaluate the efficacy of JNJ, EAE mice received 6 mg/kg (p.o.) JNJ from day 10 of immunization (disease onset) until day 42; this dose was selected based on previous studies [99,100]. The mice in the NC and EAE model groups were administered the same volume of saline. EAE was assessed daily according to the following criteria: 0, no clinical symptoms; 1, tail paralysis; 2, impaired righting reflex and partial hind limb paralysis; 3, complete hind limb paralysis; 4, hind limb paralysis with partial fore limb paralysis; and 5, death [97]. EAE scoring was performed by a trained scientist in a blinded manner. The mice were euthanized at the end of the treatment period using deep inhalational anesthesia (isoflurane). The brain and spleen were collected for subsequent molecular analyses, including flow cytometry and reverse transcription-polymerase chain reaction (RT-PCR).

### 4.4. Flow Cytometry

For the flow cytometric analysis of spleen cells, the following conjugated antibodies were used: anti-CD4 FITC, anti-CD4 PE, anti-CD4 APC, anti-CD4 PE/Dazzle, anti-IFN-γ APC/Cyanine7, anti-T-bet PE, anti-IL-9 PE, anti-IRF4 Alexa Fluor^®^ 488, anti-IL-17A PE/Dazzle, anti-RORγt PE, anti-TGF-β1 PE, and anti-Foxp3 PE. The cells were washed, and surface staining for CD4 was performed. After fixing and permeabilizing the cells, intracellular staining was performed using anti-IFN-γ, anti-T-bet, anti-IL-9, anti-IRF-4, anti-IL-17A, anti-RORγt, anti-Foxp3, and anti-TGF-β1 fluorescent antibodies. Cytokines and transcription factors were determined based on the immunofluorescence characteristics of antibody-labeled cells in the lymphocyte gate. The proportions of Th1 (CD4^+^IFN-γ^+^ and CD4^+^T-bet^+^), Th9 (CD4^+^IL-9^+^, and CD4^+^IRF4^+^), Th17 (CD4^+^IL-17A^+^ and CD4^+^RORγT^+^) and Treg (CD4^+^TGF-β1^+^ and CD4^+^Foxp3^+^) cells were determined in the lymphocyte gate. Ten thousand events were acquired on a Beckman Coulter FC500 flow cytometer (Beckman Coulter, Indianapolis, IN, USA), and the results were analyzed using CXP software 1.0 [59,61].

### 4.5. Real-Time Quantitative PCR (qRT-PCR)

Total RNA was isolated from the brain according to the manufacturer’s instructions using TRIzol reagent. A NanoDrop 2000 spectrophotometer was used to measure the RNA concentration. cDNA was synthesized using a high-capacity cDNA reverse transcription kit, and RT-PCR was performed using SYBR Green master mix (Applied Biosystems). The nucleotide sequences of the primers are as follows: IFN-γ, F: 5′-AGGAAGCGGAAAAGGAGTCG-3′ and R: 5′-GGGTCACTGCAGCTCTGAAT-3′; T-bet, F: 5′- TCAACCAGCACCAGACAGAG-3′; IL-9, F: 5′-ACCAGCTGCTTGTGTCTCTC-3′ and R: 5′-CGGCTTTTCTGCCTTTGCAT-3′; IRF4, F: 5′-GGGTGCTTTCTGTTGGCTTG-3′ and R: 5′-CTGGCTTGCCAAACACTGTC-3′; IL-17A, F: 5′-GGACTCTCCACCGCAATGAA-3′ and R: 5′-GGGTTTCTTAGGGGTCAGCC-3′; RORγ, F: 5′-AGTGTAATGTGGCCTACTCCT-3′ and R: 5′-GCTGCTGTTGCAGTTGTTTCT-3′; TGF-β1, F: 5′-ACTGCAAGTCAGAGACGTGG-3′ and R:5′-CATAGATGGCGTTGTTGCGG-3′; Foxp3, F: 5′-GGTATATGCTCCCGGCAACT-3′ and R: 5′-CACTGCCCTGAGTACTGGTG-3′; GAPDH, F: 5′-GGCAAATTCAACGGCACAGT-3′ and R: 5′-TGAAGTCGCAGGAGACAACC-3′. The relative expression levels of mRNA were calculated using the 2^−∆∆Ct^ method, and GAPDH was used as the endogenous control [59,61,101].

### 4.6. Statistical Analysis

Statistical analyses were performed using GraphPad Prism 5.0 software (GraphPad, San Diego, CA, USA). Data are presented as mean ± SD (data from six animals). The parameters were first analyzed for homogeneity and normality of variances using Bartlett’s and Kolmogorov-Smirnov tests, respectively, and found to be usually distributed and homogeneous. The results were then analyzed using parametric tests, Student’s *t*-test, and one-way ANOVA, followed by Tukey–Kramer for multiple comparisons. Statistical significance was set at *p* < 0.05.

## Data Availability

All data presented in this study are available on reasonable request from the corresponding author.

## Supplementary Materials

The supporting information can be downloaded at: https://www.mdpi.com/article/10.3390/ijms242015273/s1.
